# Intralesional microbial community signatures across histopathologic categories of tongue lesions

**DOI:** 10.3389/fmicb.2026.1775141

**Published:** 2026-05-13

**Authors:** Karthik Madhavan, Emily Lanzel, Shareef M. Dabdoub, Ahmed Sidahmed, John Hellstein, David Ray Drake, Jeffrey A. Banas, Sukirth M. Ganesan

**Affiliations:** 1 Department of Periodontics, College of Dentistry, University of Iowa, Iowa City, IA, United States; 2 Department of Oral Pathology, Radiology, and Medicine, College of Dentistry, University of Iowa, Iowa City, IA, United States; 3 Division of Biostatistics and Computational Biology, College of Dentistry, University of Iowa, Iowa City, IA, United States; 4 College of Dentistry, Iowa Institute for Oral Health Research, University of Iowa, Iowa City, IA, United States; 5 Department of Pediatric Dentistry, College of Dentistry, University of Iowa, Iowa City, IA, United States

**Keywords:** epithelial dysplasia, intralesional microbiome, microbial dysbiosis, oral premalignant lesion, oral squamous cell carcinoma

## Abstract

**Background:**

The tissue-resident microbiome of oral epithelial lesions remains incompletely characterized, with most prior studies relying on saliva or surface sampling. This study aimed to characterize intralesional microbial communities across histopathologic categories of tongue lesions using formalin-fixed paraffin-embedded (FFPE) tissue.

**Methods:**

This cross-sectional study characterized the intralesional microbiome of 63 formalin-fixed, paraffin-embedded tongue tissues, including fibroma (*n* = 15), low malignant potential dysplasia (LMP; *n* = 24), high malignant potential dysplasia (HMP; *n* = 24), and additional OSCC samples. Amplicon sequencing of the V3–V4 16S rRNA region was used to assess taxonomic composition, alpha and beta diversity, phylogenetic structure, predicted functional pathways (PICRUSt2), and machine-learning–based discrimination of lesion categories.

**Results:**

Microbial community profiles differed significantly across histopathologic groups. Non-dysplastic tissues exhibited higher richness and greater representation of commensal genera such as *Streptococcus*, *Rothia*, and *Veillonella*. Dysplastic tissues demonstrated reduced diversity and increased abundance of stress-adapted Proteobacteria including *Bosea*, *Novosphingobium*, *Sphingomonas*, and *Pseudomonas*. Beta diversity analyses revealed distinct community structures between fibroma, LMP, and HMP categories. Predicted functional profiles suggested differences in inferred metabolic potential, including pathways related to carbohydrate metabolism and xenobiotic degradation in dysplastic lesions. A supervised classifier demonstrated separation between groups (AUC 0.83–1.00), with several taxa contributing to classification; however, these findings should be interpreted cautiously given the sample size.

**Conclusion:**

Intralesional microbial communities differ across fibroma, dysplasia, and OSCC of the tongue in both taxonomic composition and predicted functional profiles. These findings describe lesion-associated microbial signatures within tissue and provide a foundation for future studies incorporating longitudinal designs and multi-omics approaches to clarify their biological and clinical relevance.

## Introduction

Oral squamous cell carcinoma (OSCC) is the most common malignancy of the oral cavity, accounting for approximately 90% for all oral cancers worldwide (Tan et al., 2023). It is associated with high rates of local invasion, recurrence, and metastasis, contributing to its classification as one of the most aggressive oral malignancies (Wang et al., 2013; Chamoli et al., 2021). Despite advances in surgical management, radiotherapy, and systemic treatments, prognosis varies widely by stage at diagnosis, and the overall 5-year survival rate remains approximately 56–73% (Rogers et al., 2009; Amit et al., 2013; Luryi et al., 2015; Garzino-Demo et al., 2016). A major clinical challenge lies in the fact that a substantial portion of OSCC cases arise from clinically identifiable precursor lesions, most commonly oral leukoplakia. Epidemiologic studies estimate that leukoplakia accounts for approximately 36% of OSCCs (Mehanna et al., 2009; Warnakulasuriya and Ariyawardana, 2016; Chaturvedi et al., 2019). These lesions often exhibit epithelial dysplasia, which is histologically graded into categories reflecting increasing malignant risk (IARC Working Group on the Evaluation of Cancer-Preventive Interventions, 2023; Hankinson et al., 2025). Although dysplasia is widely used for risk assessment, the biological factors that distinguish lesions of differing malignant potential remain incompletely understood (Speight et al., 2018a; Yang et al., 2018).

The etiology of OSCC is multifactorial and includes well-established risk factors such as use of tobacco, alcohol consumption, betel quid chewing, and chronic mucosal irritation (Neville and Day, 2002). However, these exposures alone do not fully explain why only a subset of leukoplakic or dysplastic lesions are associated with carcinoma nor do they account for the heterogeneity in clinical behavior observed among lesions with histopathological features (Speight et al., 2018b). This gap highlights the need to identify additional biological factors that may influence lesion-associated tissue environments.

Increasing attention has focused on the oral microbiome as a potential contributor to epithelial biology and disease. The oral cavity harbors more than 700 microbial species organized into site specific communities shaped by surface characteristics, oxygen tension, and host factors (Dewhirst et al., 2010; Willis and Gabaldón, 2020). These microbial communities interact closely with the oral epithelium through inflammatory signaling, immune modulation, nutrient metabolism, and biofilm-associated virulence mechanisms (Kleinstein et al., 2020) Dysbiosis, which refers to a shift in the microbial balance towards pro-inflammatory or stress adapted microorganisms, has been implicated in periodontal disease, systemic inflammation, autoimmune conditions, and oncogenesis (Khor et al., 2021).

Although altered microbial composition has been reported in OSCC (Crispino et al., 2024), the characteristics of the microbiome in earlier lesion categories, particularly epithelial dysplasia, remains poorly understood. Dysplasia represents the earliest histologic stage at which malignant risk can be estimated, and microbial features associated with dysplastic lesions may provide biologically relevant context for lesion classification. Several studies have investigated microbial communities in potentially malignant oral disorders (Herreros-Pomares et al., 2023; Pignatelli et al., 2024; Deo et al., 2025; Amer et al., 2017) reported enrichment of anaerobic and potentially pathogenic taxa such as *Fusobacterium*, *Leptotrichia*, *Campylobacter*, and *Rothia*, accompanied by depletion of commensal taxa, *Firmicutes*.

A fundamental limitation of many oral microbiome studies is their reliance on saliva-based or surface swab sampling (Heng et al., 2022). Saliva contains a composite microbial signal derived from multiple oral niches, including the buccal mucosa, gingival crevices, dorsum of the tongue, dental plaque and oropharynx. As a result, saliva-based approaches may obscure lesion-specific microbial signatures. Prior studies have demonstrated pronounced spatial heterogeneity across oral sites, driven by differences in epithelial keratinization, surface topography, salivary flow, and oxygen gradients (de Paiva et al., 2016; Mukherjee et al., 2017). Because dysplasia and OSCC arise within discrete epithelial compartments, lesion-adjacent or whole-mouth sampling may fail to capture biologically relevant microbial communities localized within the lesion microenvironment.

In contrast, tissue-resident microbiota embedded within epithelium and stroma are increasingly recognized as integral components of tumor-associated microenvironments. Studies across multiple cancer types have shown that tumors harbor microbial signatures distinct from adjacent non-malignant tissue and that these microbes may correlate with immune infiltration and inflammatory states (Nejman et al., 2020; Pratap Singh et al., 2023; Chen et al., 2024; Ma et al., 2025) further demonstrated that tissue-resident microbial DNA and circulating microbial nucleic acids can distinguish among cancer types, suggesting the presence of cancer-specific microbial signatures. Although these studies focused primarily on non-oral malignancies, similar principles may apply to oral epithelial lesions, where tissue-associated microbes may influence local inflammatory, metabolic, or signaling environments in ways not detectable by superficial sampling.

Mechanistic studies suggest that dysbiosis may influence carcinogenesis through pathways involving chronic inflammation, oxidative DNA damage, epithelial barrier disruption, cytokine induction, metabolic reprogramming, and immune modulation (Garrett, 2015). Evidence from oral microbiome research further indicates that dysbiotic communities can shape local immune responses and inflammatory microenvironments (Lamont et al., 2018). Simultaneously, loss of health-associated genera such as *Streptococcus* and *Veillonella* has been reported in dysplastic and malignant oral tissues (Zhang et al., 2020), and such changes have been linked to reduced short-chain fatty acid levels (Nouri et al., 2023). These observations support the rationale for directly examining microbial communities within lesion tissue rather than relying on whole-mouth or saliva-derived profiles.

To address these gaps, the present study employs a site-controlled, tissue-based design focused exclusively on tongue lesions. Using Formalin Fixed Paraffin embedded tissue (FFPE) specimens, we characterized intralesional microbial communities across non-dysplastic fibroma, low malignant potential (LMP) dysplasia, high malignant potential (HMP) dysplasia, and OSCC. By integrating diversity analyses, taxonomic profiling, predicted functional interference and supervised machine-learning models, this study provides a comprehensive assessment of lesion-associated microbial patterns.

The current study is novel in its use of tissue-based microbiome profiling to distinguish dysplasia subcategories within a single oral sub-site (tongue only), thereby minimizing ecological confounding. In addition, the integration of machine-learning classification enables identification of microbial features associated with specific histopathologic categories. Rather than implying temporal progression, the study characterizes distinct microbial states associated with each lesion type, providing framework for future mechanistic and longitudinal investigations.

## Methodology

### Study design and ethical approval

This study utilized a cross-sectional study using formalin-fixed paraffin-embedded (FFPE) tongue tissue specimens from the surgical pathology archives of the University of Iowa College of Dentistry Surgical Oral Pathology Laboratory. All samples were fully de-identified before proceeding analysis, and no patient contact occurred. Ethical approval was obtained from the University of Iowa, Institutional Review Board (Hawk IRB: 202010250).

### Sample collection and case selection

Formalin-fixed paraffin-embedded (FFPE) tissue of tongue lesions was retrieved from archived biopsy specimens. All samples were derived from lesions located on the tongue to minimize anatomical variability in microbial composition. Low malignant potential (LMP) and high malignant potential (HMP) dysplasia were classified using the World Health Organization (WHO) two-tier system as described in the 2017 WHO Classification of Head and Neck Tumors (Warnakulasuriya et al., 2021). All cases were independently evaluated by two board-certified oral pathologists (EL, JH) using established architectural and cytologic criteria. In cases of discordance, slides were jointly reviewed and a consensus diagnosis was reached. Control cases were similarly reviewed and confirmed as fibromas with histologically normal overlying epithelium. Demographic and clinical variables, when available, were extracted from biopsy submission records.

### DNA extraction and sequencing

Genomic data was extracted using the FFPE Tissue kit (Qiagen, Valencia, CA, USA). Paraffin was removed using Xylene and ethanol washes prior to proteinase K digestion. DNA yields were quantified using fluorometric methods, and extracts were evaluated for the presence of amplifiable bacterial DNA using 16S rRNA control primers. Targeted amplification of the 16S rRNA gene in the V3-V4 hypervariable regions was performed using forward and reverse primers, respectively:

Forward: 5′TCGTCGGCAGCGTCAGATGTGTATAAGAGACAGCCTACGGGNGGCWGCAG, Reverse: 5′GTCTCGTGGGCTCGGAGATGTGTATAAGAGACAGGACTACHVGGGTATCTAATCC.

Amplicon sequences were processed using the Illumina MiSeq Reagent Kit V3 for the Illumina MiSeq platform using 2 × 300bp paired-end sequencing.

### Contamination assessment and mitigation

Given the low-biomass nature of FFPE-derived samples, multiple steps were implemented to assess and mitigate potential environmental and reagent contamination. Negative extraction controls and reagent blanks were sequenced alongside biological samples. Amplicon sequence variants (ASVs) detected in negative controls were removed prior to downstream analysis.

In addition, taxa previously reported as potential contaminants in low-biomass microbiome studies (e.g., *Sphingomonas*, *Ralstonia*, *Pseudomonas*) were evaluated for their distribution across samples. These taxa were retained only when they demonstrated consistent patterns across biological samples and were not enriched in negative controls. Taxa not typically associated with the oral cavity were interpreted cautiously and not emphasized in downstream biological interpretation.

### Sequence processing and taxonomic assignment

Raw sequencing reads were processed using the standard DADA2 pipeline. Quality filtering, denoising, and chimera removal were performed to generate amplicon sequence variants ASVs. To reduce noise from low-prevalence features, taxa with relative abundance <10^−4^ were excluded from downstream analyses. Rarefaction was not performed, as normalization and compositional data approaches were used, consistent with current recommendations for microbiome analysis (McMurdie and Holmes, 2014; Weiss et al., 2017). ASVs were taxonomically annotated to genus-level with the QIIME2 (Bolyen et al., 2019) q2-feature-classifier plugin (Bokulich et al., 2018) using a naive Bayes machine-learning classifier trained on the full-length 16S sequences of the Human Oral Microbiome Database (HOMD) v15.4. A midpoint-rooted phylogenetic tree was created using the QIIME2 phylogeny plugin align-to-tree-mafft-fast tree command.

### Alpha diversity analysis

Alpha diversity metrics were computed to evaluate the richness and evenness across the four groups. Alpha diversity calculation, comparison and visualization was determined using PhyloToAST v1.4 (Dabdoub et al., 2016). Diversity and richness were assessed using the Shannon and abundance-based coverage estimator (ACE) metrics, and group-wise significance was determined with the Kruskal-Wallis non-parametric test. Cumulative Sum Scaling (CSS) (Paulson et al., 2013) normalization was applied to the count data prior to Bray–Curtis dissimilarity calculation using the QIIME2 beta plugin and group/pairwise comparisons.

### Beta diversity analysis

To examine between-group differences in overall community structure, beta diversity was assessed using the Bray–Curtis index. PERMANOVA comparisons were performed using the beta-group-significance plugin. Multivariate ordination of the beta diversity pairwise distances was performed using Linear Discriminant Analysis with the PhyloToAST LDA.py command. Heatmaps of genus-level relative abundances were generated using hierarchical clustering based on Bray–Curtis distances.

### Phylogenetic visualization with iTOL

To examine the taxonomic patterns at a broader phylogenetic level, normalized group-wise mean abundance was calculated using PhyloToAST’s iTol.py command and visualized on the phylogenetic tree using the EMBL iTol(Interactive Tree of Life)(v6.5.8 website) (Letunic and Bork, 2021). Circular phylograms were annotated by phylum-level taxonomic identity and colored according to relative abundance. This results in simultaneous visualization of taxonomic distribution and phylogenetic context across the lesion groups.

### Core microbiome analysis

Core microbiome analysis was performed using an 80% prevalence threshold to identify taxa consistently present within each diagnostic group. Overlap in core taxa between groups was evaluated to assess shared and unique microbial features (Neu et al., 2021).

### Functional prediction using PICRUSt2

Predicted potential functions and metabolic pathways were determined using PICRUSt2 and the KEGG Orthology (Douglas et al., 2020). Differential abundance analysis of KEGG ortholog genes was calculated using the Bioconductor packages for R: DESeq2 v1.36.0 with the variance stabilizing transformation (VST) and apeglm v1.19.0 for effect-size estimation. The resulting log2-fold changes between groups were visualized using the hierarchical circle-packing plots available through VORTEX (Gupta et al., 2017).

### Supervised machine learning classifier

A supervised decision tree classifier was constructed to identify groups of potential biomarkers that can discriminate between the three diagnostic groups. A Gradient Boosting decision tree classifier was built using the QIIME2 classify-samples command with 5-fold cross-validation, 1,000 tree estimators, recursive feature elimination for feature selection, and automatic hyperparameter tuning using random grid search.

All statistical analyses were performed using QIIME2, PhyloToAST, and R. Nonparametric tests were employed throughout due to the skewed distributions of microbial abundance. Multiple comparison corrections were applied where appropriate. Visualizations were produced in QIIME2, R, PhyloToAST, iTOL, and VORTEX.

## Results

Sixty-three paraffin embedded tongue tissue samples were analyzed to characterize the intralesional microbial communities across histopathologic grades. Following DNA extraction and quality control, samples meeting the sequencing thresholds were retained for downstream analysis. Demographic information was available for sixty-three cases, comprising of 15 fibromas (non-dysplastic control), 24 lesions with low malignant potential (LMP), and 24 with high malignant potential (HMP). Fourteen additional oral squamous cell carcinoma (OSCC) tissues were included for comparative analyses but were not included in the demographic table as the corresponding clinical metadata was not available. All lesions were confined to the tongue to minimize site-specific variation in microbial composition.

Age varied significantly among the three diagnostic groups (ANOVA *p* = 0.014). Tukey HSD pairwise comparisons revealed that participants with LMP lesions were older on average than participants with HMP lesions (mean difference = 9.78 years, *p* value = 0.003) and Fibroma (mean difference = 11.24 years, p value = 0.0334), whereas there were no differences detected between fibroma and HMP (p value = 0.9391). Sex distribution across the study also differed significantly across the groups (Fisher’s exact test *p* = 0.04), with males being more prevalent in the LMP and HMP cohorts compared with the fibroma group. Demographic details are summarized in Table 1.

**Table 1:** Demographic characteristics of the study cohort.

Variable	Fibroma (*n* = 15)	LMP (*n* = 24)	HMP (*n* = 24)	Total (*n* = 63)	*p*-value
Age (years), Median	51	63	52	58	0.01 (ANOVA)
Age (years), range	26–74	40–88	32–80	26–88	
Male *n* (%)	6 (40%)	14 (56%)	15 (63%)	35 (55%)	0.04 (Fisher exact test)
Female *n* (%)	9 (60%)	11 (44%)	9 (37%)	29 (45%)	

A total of 2.73 million high-quality sequencing reads were generated from 63 samples. After quality filtering and trimming, a median of 70,900 reads per sample (SD = 25,910) was retained. Across all datasets, 2,439 amplicon sequence variants ASVs corresponding to 139 bacterial genera were identified. Read depth and quality metrics were consistent across diagnostic groups, providing uniform coverage for comparative analyses.

### Alpha and beta diversity patterns

**Figure 1:**
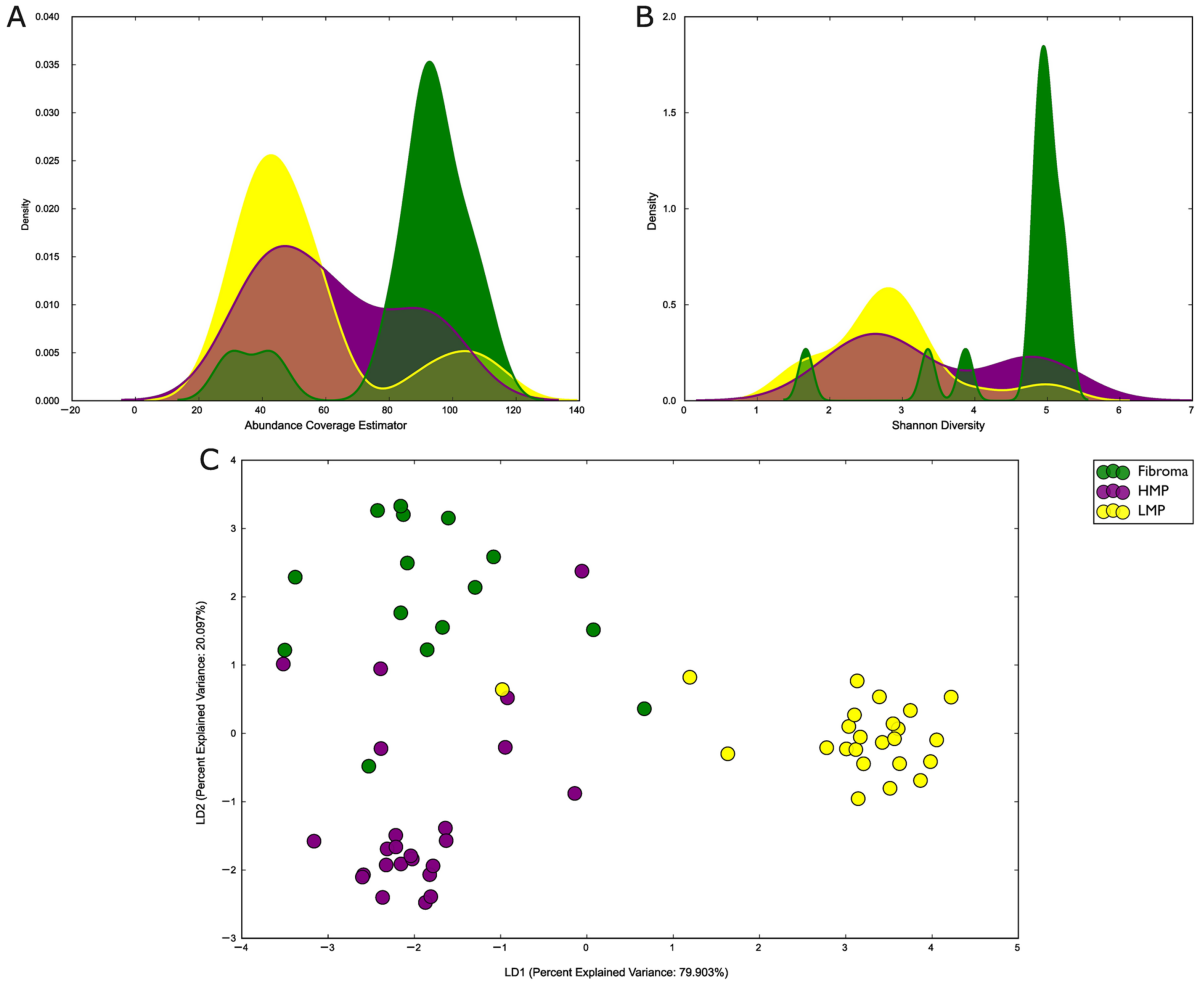
**(A)** ACE richness across fibroma, LMP, and HMP groups. **(B)** Shannon diversity across fibroma, LMP, and HMP groups. **(C)** Bray–Curtis linear discriminant analysis (LDA) plot illustrating clear separation of microbial communities across normal, LMP, and HMP groups.

Alpha diversity metrics demonstrated differences in microbial richness and evenness across histopathologic categories. ACE richness values were highest in fibromas, intermediate in the LMP, and lowest in HMP, indicating reduced representation of low-abundance taxa in dysplastic lesions (Figure 1A). Similarly, Shannon diversity was lower in HMP relative to fibroma, reflecting reduced community evenness (Figure 1B).

Beta diversity analysis using the Bray–Curtis dissimilarity further demonstrated a distinct microbial community structure across diagnostic groups (Figure 1C). Ordination plots showed separation between fibroma, LMP, and HMP samples, with partial overlap between groups. Pairwise PERMANOVA testing confirmed significant differences in community composition between fibroma and HMP (*p* = 0.001), fibroma and LMP (p = 0.001), and LMP and HMP (*p* = 0.003). These findings indicate that microbial community composition differs across histopathologic categories. To assess potential confounding by demographic variables, additional PERMANOVA analyses were performed evaluating age and sex as independent factors. No significant differences in overall microbial composition were observed based on age (*p* = 0.404) or sex (*p* = 0.288). These findings suggest that the observed differences in microbial community structure across histopathologic categories are unlikely to be driven solely by these variables.

### Phylogenetic context of community shifts

**Figure 2:**
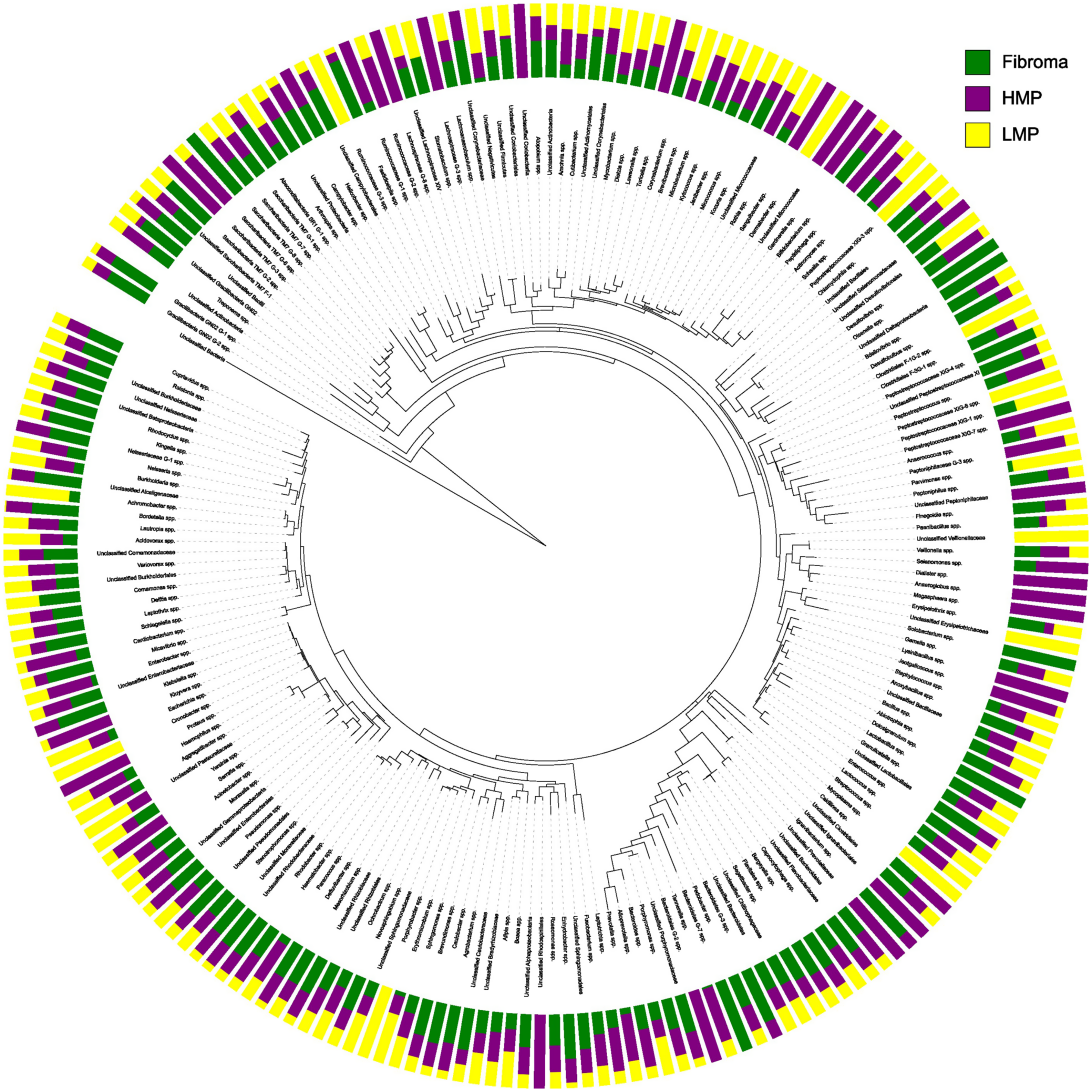
Phylogenetic distribution of intralesional microbiome abundance. Circular phylogenetic tree (iTOL) illustrating relative abundance of major bacterial phyla and associated genera in Fibroma, LMP, and HMP lesions.

The circular iTOL phylogenetic tree illustrated the distribution of dominant taxa across diagnostic categories (Figure 2). Fibroma displayed a community enriched in commensal *Firmicutes* and *Actinobacteria*, including *Streptococcus, Rothia,* and *Veillonella*. In contrast, both LMP and HMP lesions showed increased representation of Proteobacteria, including *Bosea, Sphingomonas, Novosphingobium,* and *Pseudomonas*. The tree highlights in community composition, with reduced representation of commensal taxa and increased relative abundance of *Proteobacteria* in dysplastic lesions.

### Machine learning classifier

**Figure 3:**
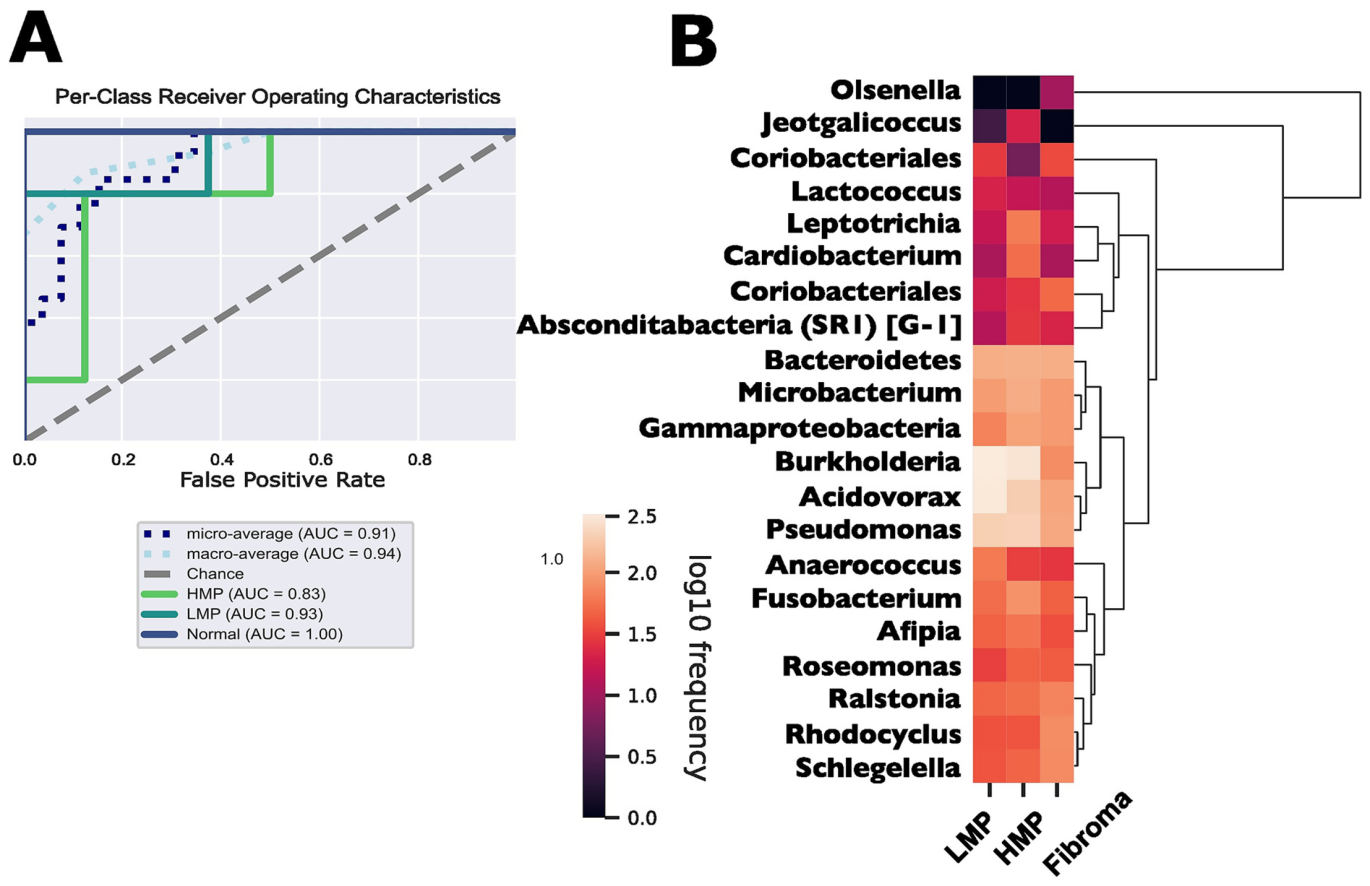
**(A)** Receiver operating characteristic (ROC) curves showing classifier performance for fibroma, LMP, and HMP lesions. Micro- and macro average AUC values are indicated. **(B)** Genus-level heatmap of intralesional microbiome. Heatmap of top-ranked features contributing to classification, based on feature importance scores from the gradient boosting model. The classifier was trained using an 80/20 train–validation split with 5-fold cross-validation. Feature importance reflects contribution to classification within this dataset.

A supervised gradient boosting classifier trained on the genus-level abundance profiles, distinguished fibroma, LMP, and HMP groups with a micro-average AUC of 0.91 and macro-average AUC of 0.94 (Figure 3A). The most influential features included *Bosea*, *Novosphingobium*, *Sphingomonas*, and *Pseudomonas* (Figure 3B). These taxa were also identified in differential abundance analyses, indicating that they contribute to classification of histopathologic categories. However, given the cross-sectional design, these features should be interpreted as discriminatory taxa associated with lesion categories rather than predictive biomarkers of malignant transformation.

### Predicted functional potential reflects metabolic and inflammatory remodeling

**Figure 4:**
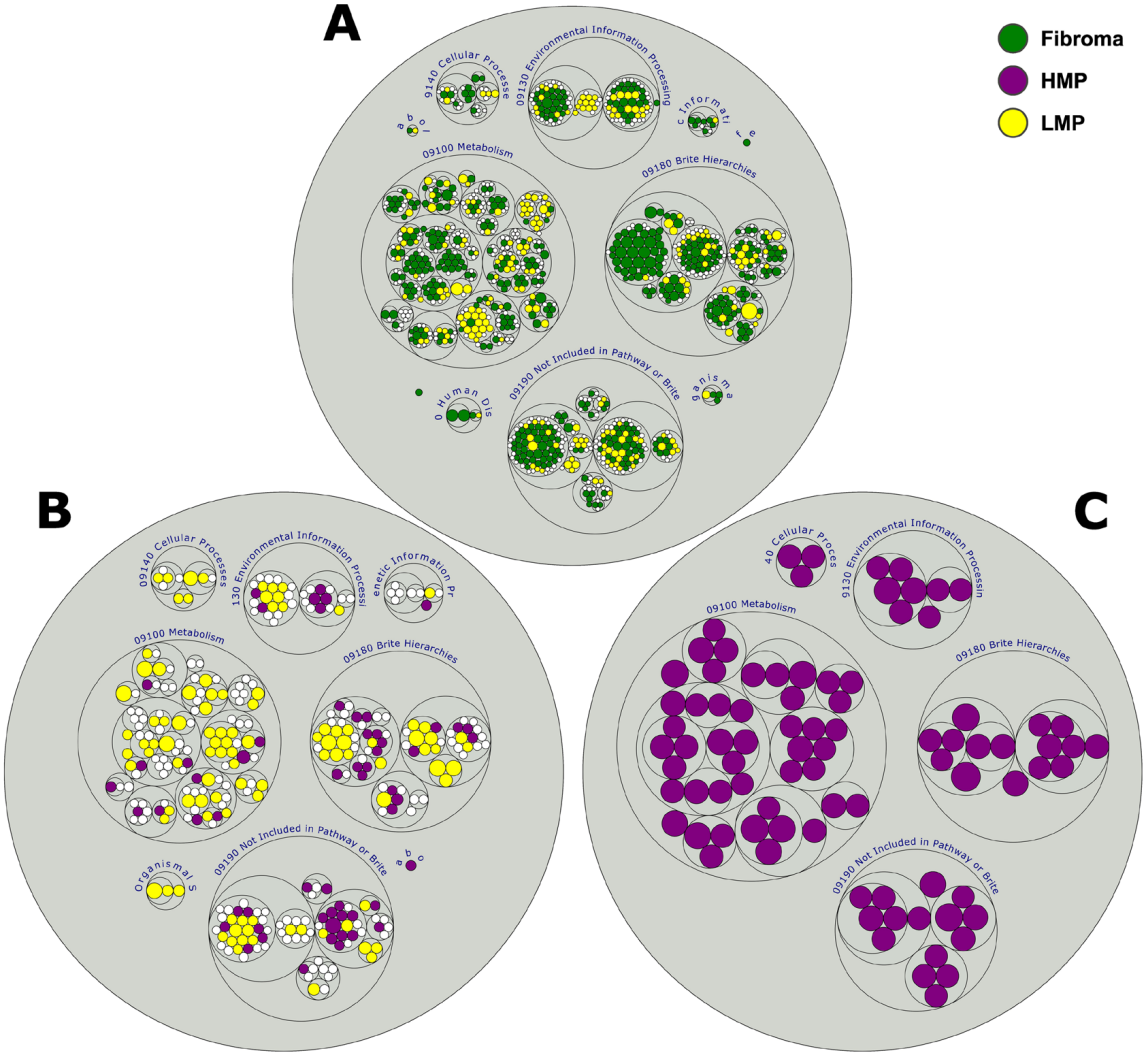
PICRUSt2-predicted microbial functions across lesion categories. **(A)** Fibroma vs. LMP, **(B)** LMP vs. HMP, **(C)** Fibroma vs. HMP. Each panel shows differential enrichment of KEGG Level 2 pathways; colors correspond to functional pathways enriched in the corresponding histopathologic categories. Functional profiles were inferred using PICRUSt2 and represent predicted metabolic potential based on 16S rRNA gene data. These predictions should be interpreted as hypothesis-generating.

Functional metagenomic inference using the PICRUSt2 revealed distinct predicted functional profiles across groups. Dysplastic lesions showed higher relative representation of pathways related to carbohydrate metabolism, xenobiotic degradation, and lipopolysaccharide biosynthesis, whereas fibroma samples showed greater representation of biosynthetic pathways (Figures 4A–C). These predictions are based on 16S inference and should be interpreted as indicative of potential functional differences rather than direct evidence of metabolic activity.

### Core microbiome: stability loss with advancing lesion grade

**Figure 5:**
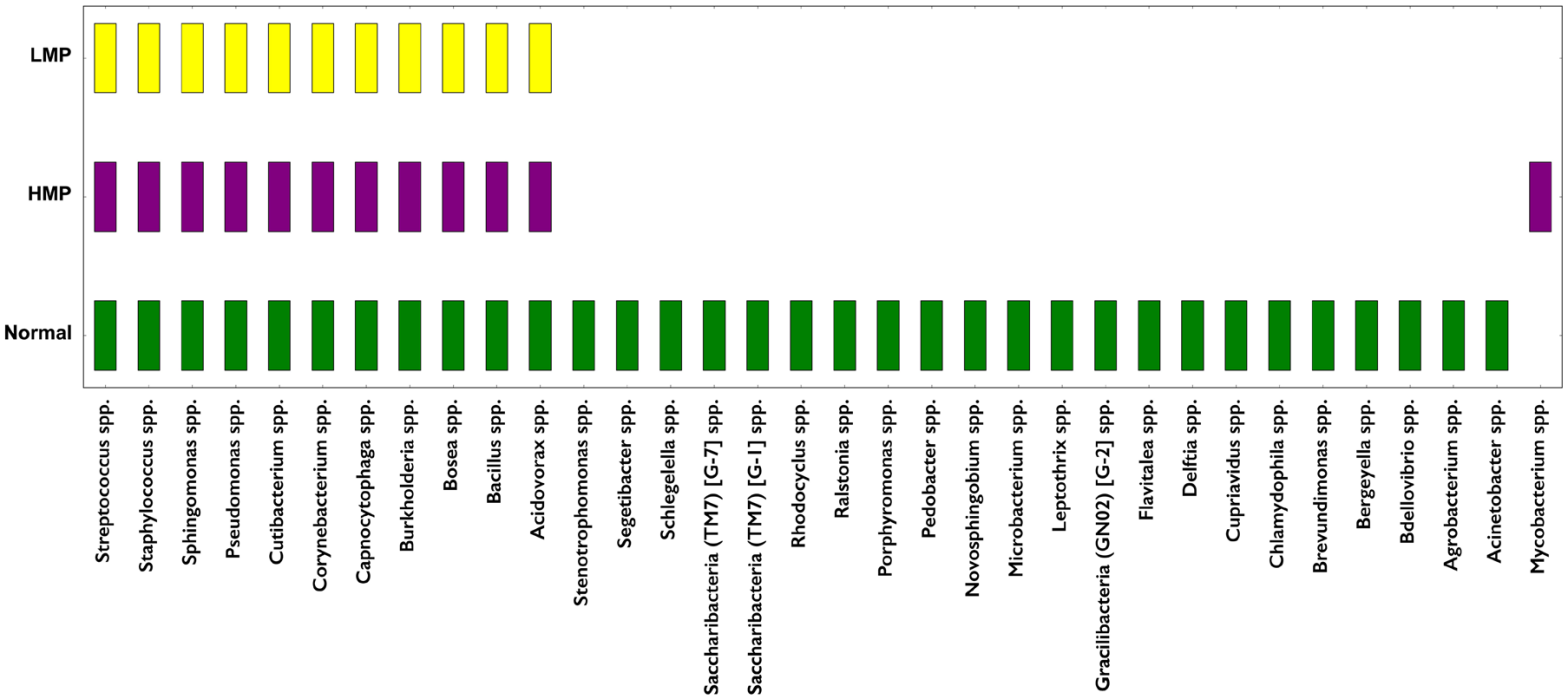
Core microbiome analysis across histopathologic categories. Core microbiome analysis at an 80% prevalence threshold showing shared and unique taxa across fibroma, LMP, and HMP groups. Venn diagram illustrates overlap in core genera between groups. The analysis demonstrates differences in shared core taxa across diagnostic categories, with reduced overlap between fibroma and HMP.

Core microbiome analysis at an 80% prevalence threshold demonstrated a substantial reduction in shared taxa across different lesion grades (Figure 5). Fibroma and LMP groups shared about 80% of dominant genera, whereas there was an overlap between Fibroma and HMP, which decreased to ~60% (Figure 5). Core genera common to Fibroma included *Streptococcus*, *Rothia*, and *Veillonella*. In dysplastic lesions, these taxa were replaced by Proteobacterial members such as *Bosea*, *Pseudomonas*, and *Sphingomonas*. The reduced overlap in core taxa highlights differences in community composition across diagnostic categories.

## Discussion

This study provides a single-site, tissue-based characterization of the intralesional microbiome across non-dysplastic, dysplastic, and malignant tongue lesions using FFPE-derived samples. By examining microbial communities within the epithelial microenvironment itself, the analysis offers insight into microbial composition and predicted functional profiles associated with distinct histopathologic categories. As this is a cross-sectional study, the observed differences represent lesion-associated microbial signatures rather than temporal or causal transitions. Nevertheless, defining these category-specific ecological patterns is important for understanding the microenvironment in which epithelial alterations are identified.

While a large body of literature has focused on microbiomes in systemic and organ-specific cancers, including the gut, breast, and colorectal sites (Wang et al., 2023; Neagoe et al., 2024; Nobels et al., 2025), studies evaluating intralesional microbial communities within the oral cavity remains relatively limited. This work contributes to that gap by characterizing the intra-tissue microbiome of tongue lesions across benign, dysplastic, and malignant lesions.

Across diagnostic groups, non-dysplastic fibromas demonstrated greater microbial richness and higher representation of health-associated genera such as *Streptococcus*, *Rothia*, and *Veillonella*. In contrast, dysplastic lesions demonstrated communities enriched for Proteobacteria, including *Bosea*, *Novosphingobium*, *Sphingomonas*, and *Pseudomonas*. These findings are consistent with previous tissue-based studies reporting reduced abundance of commensal taxa and increased abundance of stress-tolerant, oxidative-adapted organisms in oral epithelial lesions (Zeng et al., 2022; Radaic et al., 2023; Wright et al., 2023; Pratap Singh et al., 2023). The present results reinforce that microbial signatures differ markedly between non-dysplastic, dysplastic, and malignant tissue categories and maybe associated with local microenvironmental conditions such as oxidative stress, nutrient flux, or inflammatory signaling (Liu and Zhang, 2022).

Predicted functional profiles generated using PICRUSt2 suggested differences in inferred metabolic potential across lesion categories. Dysplastic lesions showed higher representation of pathways related to carbohydrate metabolism, xenobiotic degradation, and lipopolysaccharide biosynthesis pathways, whereas non-dysplastic controls exhibited higher representation of amino acid and nucleotide biosynthesis pathways. These predicted functions should be interpreted as hypothesis-generating given the limitations of 16S-based inference. Integration with metatranscriptomic or metabolomic approaches will be necessary to determine whether these predicted pathways are functionally active within dysplastic lesions.

The enrichment of xenobiotic degradation pathways is notable, as oral bacteria are capable of metabolizing exogenous carcinogens such as nitrosamines, polycyclic aromatic hydrocarbons, and acetaldehyde (Meurman, 2010). Similarly, the predicted enrichment of carbohydrate metabolism and lipopolysaccharide biosynthesis pathways in dysplastic lesions is consistent with prior tumor–microbiome studies describing microbial adaptation within altered epithelial environments (Ciernikova et al., 2022). However, these observations should be interpreted cautiously, as functional predictions do not capture gene expression or host–microbe interactions.

The supervised machine-learning classifier demonstrated separation between diagnostic groups, and several genera identified as top discriminant features, such as *Bosea* and *Novosphingobium*, were also highlighted in the differential abundance analysis. While these results indicate that the intralesional microbiome contains informative signatures associated with each lesion category, model performance should be interpreted cautiously due to sample size and the potential risk of overfitting. Larger, prospectively collected independent datasets are required to assess reproducibility, generalizability, and robustness.

A key strength of this study is the restriction to lesions located exclusively on the tongue, which minimizes anatomical heterogeneity, a major confounder in oral microbiome studies. At the same time, this design limits generalizability to other subsites of the oral cavity that harbor distinct microbial ecologies. Additional limitations include the use of FFPE-derived DNA, which may introduce bias due to DNA fragmentation and increased susceptibility to environmental or reagent contamination. Although negative controls and filtering strategies were implemented, taxa commonly reported in low-biomass or reagent-associated contexts should be interpreted with caution. Age and sex differed across diagnostic groups and may contribute to variation in the oral microbiome. In this study, exploratory analyses did not identify significant differences in overall microbial composition based on age (*p* = 0.404) or sex (*p* = 0.288). However, given the cross-sectional design and sample size, residual confounding cannot be excluded.

Furthermore, behavioral variables such as smoking, alcohol use, and oral hygiene practices were not consistently available and may contribute to observed microbial variation.

Overall, the study demonstrates that tissue-associated microbial communities differ significantly across fibroma, dysplasia, and OSCC of the tongue, both in taxonomic composition and predicted functional profiles. These findings highlight the importance of evaluating tissue-resident microbial communities and provide a foundation for future studies integrating longitudinal sampling, multi-omics approaches, and host-response data to better understand the role of the intralesional microbiome in oral epithelial pathology.

## Conclusion

This study demonstrates that tongue lesions across different histopathologic categories are associated with distinct tissue-resident microbial communities, characterized by differences in taxonomic composition, community diversity, and predicted functional profiles. Non-dysplastic, dysplastic, and malignant tissues exhibited variation in the relative representation of commensal taxa and Proteobacteria, as well as differences in inferred metabolic pathways. These findings indicate that the intralesional microbiome differs across lesion categories and may reflect aspects of the local tissue microenvironment.

Given the cross-sectional design, these observations represent associations and do not imply temporal or causal relationships. While the identified microbial patterns may provide additional context for understanding lesion-associated environments, their clinical relevance remains to be determined. Future studies incorporating longitudinal sampling and multi-omics approaches, including metatranscriptomics, metabolomics, and host-response profiling, will be necessary to define the functional activity of these microbial communities and to evaluate their potential role in oral epithelial pathology.

## Data Availability

The datasets presented in this study can be found in online repositories. The names of the repository/repositories and accession number(s) can be found at: https://www.ncbi.nlm.nih.gov/, PRJNA1393406.
